# A Life of Accidents

**Published:** 1844-08

**Authors:** 


### A LIFE OF ACCIDENTS.

A correspondent, in a recent letter to us, speaking of an accident that had befallen a common friend, gives the following little bit of history:

"He has now had a white-swelling, has had his leg broken, his thigh broken, his right arm broken, his left shoulder dislocated, his right eye put out, and one-third of the retina of the left paralyzed, and has been *snake-bitten.*"

Our correspondent asks, very gravely, "what do you think of that?" We repeat the question to the reader, only adding that the gentleman to whom it refers is an extensive merchant, is the father of a large family, is a most estimable and useful man, and bids fair to live yet many years, C.

